# Post-stroke anxiety symptoms moderate the predictive efficacy of MIP1-α on acute ischemic stroke severity

**DOI:** 10.3389/fpsyt.2026.1785971

**Published:** 2026-10-21

**Authors:** Mingzhe Li, Tengyue Chen, Yan Wang, Minjie Zhou, Xinyi Zhou, Lifei Xing, Hong Huang, Peipei Zhou, Zhonglu Liao, Yanxiang Chang, Xuan Hu, Xingguang Luo, Yu-Hsin Chen, Li Chen, Yanlong Liu, Wei Wang, Fan Wang, Huilan Jiang

**Affiliations:** 1 School of Mental Health, Wenzhou Medical University, Wenzhou, China; 2 Department of Neurology, Sinopharm North Hospital, Baotou, China; 3 College of Public Health, Wenzhou Medical University, Wenzhou, China; 4 Department of Psychiatry, Yale University School of Medicine, New Haven, CT, United States; 5 The Affiliated Kangning Hospital, Wenzhou Medical University, Wenzhou, China; 6 Wenzhou Key Laboratory of Basic and Translational Research for Mental Disorders, Wenzhou Medical University, Wenzhou, China; 7 Beijing Hui-Long-Guan Hospital, Peking University, Beijing, China; 8 Health Development Department, The Third Hospital of Quzhou, Quzhou, Zhejiang, China

**Keywords:** generalized anxiety disorder, ischemic stroke, MIP-1α, moderation, neuroinflammation

## Abstract

**Background:**

Ischemic stroke (IS) elicits a robust neuroinflammatory response in which damage-associated molecular patterns (DAMPs) released from necrotic tissue drive systemic cytokine activation. Although macrophage inflammatory protein-1α (MIP-1α) is a recognized biomarker of stroke severity, its interaction with neuropsychiatric comorbidities, particularly Post-Stroke Anxiety symptoms (PSAS), remains poorly understood. This study examined whether anxiety symptom severity moderates the association between MIP-1α levels and acute stroke severity.

**Methods:**

A total of 263 patients with mild-to-moderate ischemic stroke were enrolled. Stroke severity was assessed using the National Institutes of Health Stroke Scale (NIHSS), and anxiety symptoms were evaluated using the Generalized Anxiety Disorder-7 (GAD-7) scale. To explore potential biomarker-specific pathways, three CC chemokines (MCP-1, MIP-1α, and MIP-1β) were measured. Because primary correlation screens indicated a strong differential profile specifically for MIP-1α across anxiety severity groups, subsequent moderation analyses were focused on MIP-1α as an exploratory, hypothesis-generating objective. Moderation effects were tested using hierarchical linear regression models adjusted for age, sex, and lifestyle factors. Predictive performance was evaluated using receiver operating characteristic (ROC) curve analysis and DeLong’s test.

**Results:**

Hypothesis-generating analysis revealed a significant interaction between MIP-1α and anxiety symptom severity on NIHSS scores (standardized β = 0.16, p < 0.01). Among patients with lower anxiety (GAD-7 ≤ 4), MIP-1α was inversely associated with stroke severity. In contrast, among patients with higher anxiety (GAD-7 > 4), this association reversed, with higher MIP-1α levels correlating with greater neurological impairment. The integrated model incorporating the MIP-1α × GAD-7 interaction had the highest AUC (0.76) and significantly outperformed Model 1 (AUC = 0.67; p <0.001), but not Model 2 (AUC = 0.74; p = 0.14).

**Conclusion:**

Anxiety symptom severity moderates the cross-sectional association between serum MIP-1α levels and acute neurological impairment in ischemic stroke. These findings indicate that the prognostic value of MIP-1α is context-dependent and highlight the importance of joint neuroinflammatory and psychological evaluation during acute stroke care. These findings suggest that anxiety symptom severity moderates the cross-sectional association between MIP-1α and acute neurological impairment. Future prospective studies are needed to clarify these relationships and evaluate whether managing acute anxiety symptoms can influence neuroinflammatory signaling or clinical recovery.

## 1 Introduction

Ischemic stroke (IS) remains a leading global cause of morbidity and mortality (1). Acute neuroinflammation is a well-established pathophysiological hallmark that drives stroke progression (2). During ischemic injury, disruption of the blood–brain barrier (BBB) increases vascular permeability, allowing damage-associated molecular patterns (DAMPs), derived from necrotic brain tissue, to enter the peripheral circulation and trigger inflammatory activation of immune cells. Peripheral inflammatory cytokines, such as macrophage inflammatory protein-1α (MIP-1α), have emerged as critical biomarkers for characterizing stroke severity (3, 4). In addition, MIP-1α plays a key role in regulating leukocyte trafficking and mediating secondary cerebral injury (5–7). Despite its recognized involvement in stroke pathology, the mediating mechanisms and contextual moderators through which MIP-1α influences long-term recovery remain poorly understood. CC-chemokines like MIP-1α have traditionally been viewed as toxic mediators of acute neuroinflammation, emerging evidence indicates that CC-chemokine signaling plays a complex, context-dependent dual role in cerebral ischemia, alternating between adaptive neuroplasticity support and destructive inflammatory cascades depending on the pathological microenvironment (8). We aimed to explore whether anxiety symptom severity moderates the cross-sectional association between serum MIP-1α levels and acute neurological impairment during the early hospitalization phase.

Within the framework of psychoneuroimmunology, accumulating evidence has established biological links between cytokine dysregulation and anxiety, suggesting that pro-inflammatory mediators can directly precipitate anxiety-like behaviors via modulation of neural circuitry (9, 10). Post-Stroke Anxiety Symptoms(PSAS), one of the most prevalent neuropsychiatric sequelae, affects approximately 20–25% of stroke survivors (11). PSA not only markedly impairs functional recovery but also substantially diminishes long-term quality of life (12). A recent longitudinal cohort study of mild-to-moderate stroke patients demonstrated that acute post-stroke anxiety symptoms persist through 12 months post-event, directly exacerbating perceived functional recovery and long-term health-related quality of life (13).In a 2025 observational cohort of acute ischemic stroke, baseline stroke severity independently predicted the manifestation of post-stroke anxiety symptoms, which in turn significantly correlated with prolonged hospitalization and poorer 6-month functional outcomes (14). So understanding the interaction between anxiety symptoms and neuroinflammation may provide insights into the complex acute-phase response following ischemic stroke. Increasingly, the relationship between stroke severity and PSA is conceptualized as a complex, bidirectional interaction rather than a unidirectional consequence (15). Although initial neurological severity is a well-documented predictor of anxiety, emerging evidence suggests a deleterious reverse effect whereby PSA exacerbates neurological deficits and hinders recovery, potentially through maladaptive stress responses and dysregulation of the hypothalamic–pituitary–adrenal (HPA) axis (15). Nevertheless, despite these established pairwise associations, the intrinsic tripartite interplay among the neuroinflammatory driver (MIP-1α), psychiatric status (PSA), and neurological impairment, as measured by the NIH Stroke Scale (NIHSS), remains unclear.

Critically, it is unknown whether PSA functions as a moderator, that is, a factor that amplifies inflammatory pathogenicity, thereby intensifying the impact of MIP-1α on stroke severity. Elucidating this interaction is essential for advancing mechanistic understanding of ischemic stroke and for developing targeted, multidimensional therapeutic strategies. To address this knowledge gap, the present study investigates whether anxiety symptom severity exerts a significant moderating effect on the association between MIP-1α levels and stroke severity (NIHSS scores). We hypothesize that heightened anxiety states disrupt the neuroimmune milieu, thereby altering the functional impact of MIP-1α on neurological outcomes. The findings of this study are expected to clarify the mechanistic role of post-stroke anxiety symptoms in acute stroke pathophysiology, provide condition-dependent evidence for the clinical utility of MIP-1α as a biomarker, and establish a theoretical foundation for precision interventions targeting anxiety in stroke patients.

## 2 Materials and methods

### 2.1 Participants

A total of 263 patients diagnosed with mild to moderate ischemic stroke (IS) and admitted to the Northern Hospital of Sinopharm Group between June 2020 and December 2021 were consecutively enrolled in this study. Demographic information, including age and sex, was extracted from the hospital’s electronic medical record system. Clinical data, including histories of substance use and dependence, were obtained from medical records and patient self-reports and were further verified through interviews with immediate family members or primary caregivers. Eligible participants were adult patients (≥18 years) presenting with acute mild-to-moderate ischemic stroke, defined operationally as a baseline National Institutes of Health Stroke Scale (NIHSS) score of ≤15 upon admission (with mild stroke defined as NIHSS ≤4 and moderate stroke as NIHSS 5–15). Patients with severe stroke (NIHSS >15) were excluded to minimize confounding from massive tissue necrosis and acute critical illness instability. Vascular exclusion criteria included patients with known intracranial vascular malformations, active vasculitis, or significant pre-existing cerebrovascular lesions independent of the index stroke event. These criteria were applied to prevent baseline neuroinflammatory heterogeneity and systemic vascular inflammatory signaling from confounding serum MIP-1α concentrations.

Post-stroke anxiety symptoms were assessed using the Generalized Anxiety Disorder scale (GAD-7) via self-administered questionnaires. Participants were screened according to the following inclusion criteria: (1) age between 45 and 80 years; and (2) a clinical diagnosis of mild or moderate ischemic stroke based on neurological symptoms, physical examination, and neuroimaging findings. Exclusion criteria were as follows: (1) a pre-existing or current diagnosis of anxiety disorders (including generalized anxiety disorder, panic disorder, and social anxiety disorder) prior to stroke onset; (2) severe arterial stenosis of the internal or external carotid, subclavian, or vertebral arteries confirmed by transcranial magnetic resonance angiography or color Doppler ultrasonography; (3) a history of malignancy; (4) severe or persistent neuropsychiatric disorders (other than anxiety disorders) that could confound anxiety assessment; (5) current use of medications or supplements known to induce or exacerbate anxiety symptoms (e.g., corticosteroids, certain stimulants, or psychotropic agents); (6) severe or very severe ischemic stroke, particularly cases accompanied by impaired consciousness (e.g., coma), which could compromise the reliability of self-reported anxiety assessments; (7) substance abuse or dependence; and (8) other severe neuropsychiatric disorders diagnosed according to the Diagnostic and Statistical Manual of Mental Disorders, Fifth Edition (DSM-5), that may interfere with anxiety evaluation.

### 2.2 Stroke severity assessment

Stroke severity was assessed using the National Institutes of Health Stroke Scale (NIHSS), a standardized neurological examination comprising 15 items that evaluate level of consciousness, eye movements, visual fields, facial motor function, limb strength, sensation, coordination, speech, language, and other neurological domains. Individual items are scored on ordinal scales ranging from 0–2, 0–3, or 0–4, yielding a total score between 0 and 42, with higher scores indicating greater neurological impairment.

### 2.3 Anxiety symptom severity assessment

Anxiety symptoms were evaluated using the Generalized Anxiety Disorder scale (GAD-7), which measures the severity of anxiety-related symptoms experienced over the preceding two weeks. The GAD-7 is a validated self-report screening tool designed to assess anxiety symptom severity over the preceding two weeks, rather than to establish a formal clinical diagnosis of generalized anxiety disorder. The scale assesses core dimensions of generalized anxiety, including excessive worry, restlessness, fatigue, impaired concentration, irritability, muscle tension, and sleep disturbance. The total score ranges from 0 to 21 for the GAD-7 version, which is the most widely used format in both clinical and research settings, with higher scores reflecting greater anxiety severity.

Participants were categorized into a lower anxiety symptom group (GAD-7 ≤4) and a higher anxiety symptom group (GAD-7 >4, equivalent to ≥5). This threshold represents at least mild anxiety symptoms according to the established GAD-7 severity classification and has been applied in previous studies of anxiety symptoms after acute ischemic stroke [PMID: 33841304].

### 2.4 Measurement of inflammatory factors

Peripheral venous blood samples were collected from all participants upon hospital admission. Serum was immediately separated by centrifugation and stored at −80 °C until analysis. Serum concentrations of monocyte chemoattractant protein-1 (MCP-1), macrophage inflammatory protein-1α (MIP-1α), and macrophage inflammatory protein-1β (MIP-1β) were quantified using commercially available enzyme-linked immunosorbent assay (ELISA) kits (Shanghai Xinle Biotechnology Co., Ltd., Shanghai, China), according to the manufacturer’s protocols. All laboratory personnel performing the assays were blinded to participants’ clinical characteristics and demographic information.

### 2.5 Statistical analysis

All statistical analyses were conducted using R software (version 4.2.2). Continuous variables were summarized as mean ± standard deviation (SD), while categorical variables were reported as frequencies and percentages. For the descriptive four-group comparisons, MIP-1α was dichotomized using the median within each anxiety symptom group. Continuous variables were compared across the four independent groups using the Kruskal–Wallis test, followed by Dunn’s pairwise *post-hoc* tests with Holm correction when the omnibus test was significant. Categorical variables were compared using Pearson’s χ² test. Spearman’s rank correlation coefficients were calculated to assess bivariate associations among inflammatory markers, anxiety symptoms, and stroke severity.

To examine whether anxiety symptom group moderated the association between MIP-1α and NIHSS scores, hierarchical linear regression models were constructed (PMID: 27865431, PMID: 19587209). Covariates were selected *a priori* based on their clinical relevance to neurological severity and inflammatory biomarker concentrations. Model 1 included age, sex, smoking, alcohol consumption, hypertension, and diabetes. Model 2 additionally included MIP-1αand anxiety symptom group, and Model 3 further incorporated the MIP-1α × anxiety symptom group interaction term to formally test the moderating effect.

Significant interaction effects were probed using simple slope analyses and graphically visualized. Model comparisons and overall model fit were evaluated using F-tests. All statistical tests were two-tailed, and a p-value < 0.05 was considered statistically significant (α = 0.05).

In addition, receiver operating characteristic (ROC) curve analyses were performed to evaluate the ability of the three regression models to discriminate between patients with NIHSS = 0 and those with NIHSS >0. This prespecified threshold distinguished no deficit captured by the NIHSS from at least one measurable deficit, while recognizing that NIHSS = 0 does not exclude ischemic stroke (PMID: 20828876). The areas under the ROC curves (AUCs) were compared using DeLong’s test.

## 3 Results

### 3.1 Demographic and clinical characteristics

Descriptive statistics for the four subgroups are summarized in **Table 1**. Sex distribution differed significantly across the groups (p = 0.038). Descriptively, the proportions of females were higher in the two GAD-7 >4 subgroups than in the corresponding GAD-7 ≤4 subgroups. No significant differences were observed in age, smoking, or alcohol consumption (all p > 0.05).

**Table 1:** Baseline characteristics of study participants stratified by GAD-7 (≤4 vs. >4) and MIP-1α levels (below vs. above median).

Characteristic	GAD-7 (≤4)		GAD-7 (>4)		P
	MIP-1α (<median, n= 77)	MIP-1α (≥median, n= 77)	MIP-1α (<median, n=54)	MIP-1α (≥median, n=55)	
Demographics					
Age, years	64.77 ± 8.70	65.96 ± 8.35	67.00 ± 7.53	66.35 ± 9.85	0.55
Gender					<0.05*
Male	52 (67.50%)	48 (62.30%)	27 (50.00%)	25 (45.40%)	
Female	25 (32.40%)	29 (37.60%)	27 (50.00%)	30 (54.60%)	
Active drinker					1.00
Yes	21 (27.20%)	20 (25.90%)	14 (25.90%)	14 (25.40%)	
No	56 (72.80%)	57 (74.10%)	40 (74.10%)	41 (74.50%)	
Active smoker					0.11
Yes	43 (55.80%)	39 (50.60%)	20 (37.00%)	22 (40.00%)	
No	34 (44.20%)	38 (49.40%)	34 (63.00%)	33 (60.00%)	
Clinical scores					
NIHSS total scores	1.32 ± 1.35	0.35 ± 0.66	1.54 ± 1.42	1.24 ± 1.47	<0.001***
Comorbidities					
Hypertension					0.11
Yes	42 (54.50%)	52 (67.50%)	34 (62.90%)	41 (74.50%)	
No	35 (45.50%)	25 (32.50%)	20 (37.10%)	14 (25.50%)	
Diabetes					0.52
Yes	24 (31.10%)	18 (23.30%)	12 (22.20%)	17 (30.90%)	
No	53 (68.90%)	59 (76.70%)	42 (77.80%)	38 (69.10%)	
Biochemical indicators					
MCP-1	122.25 ± 53.30	168.51 ± 75.51	117.67 ± 52.54	164.27 ± 70.52	<0.001***
MIP-1β	104.27 ± 37.63	187.18 ± 56.20	98.77 ± 32.21	179.57 ± 64.69	<0.001***

Data are presented as n (%) or mean ± SD. Continuous variables were compared using the Kruskal–Wallis test, followed by Dunn’s pairwise *post-hoc* tests with Holm correction when appropriate. Categorical variables were compared using Pearson’s χ² test. *p < 0.05, p<0.001***.

GAD-7, Generalized Anxiety Disorder Scale; NIHSS, National Institutes of Health Stroke Scale; MIP-1α, Macrophage Inflammatory Protein-1α; MCP-1, Monocyte Chemoattractant Protein-1; MIP-1β, Macrophage Inflammatory Protein-1β.

NIHSS scores differed significantly across the four groups (Kruskal–Wallis H = 36.51, p < 0.001; **Figure 1**). Dunn’s *post-hoc* tests with Holm correction showed that the GAD-7 ≤4/MIP-1α ≥median group had significantly lower NIHSS scores than the other three groups (all adjusted p < 0.001), whereas the remaining pairwise comparisons were not significant. Hypertension and diabetes did not differ significantly across the groups (both p > 0.05).

**Figure 1:**
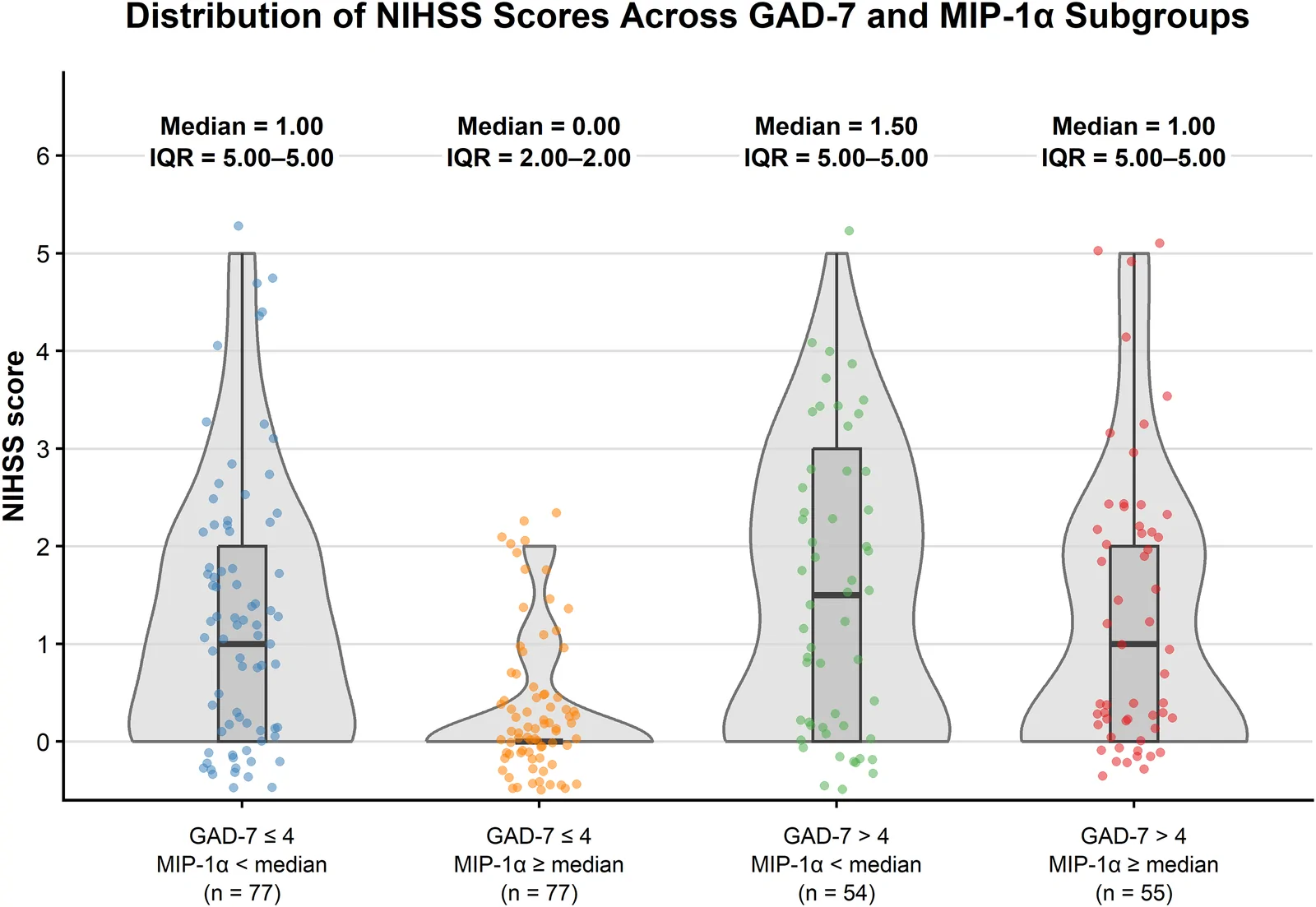
Distribution of NIHSS scores across GAD-7 and MIP-1α subgroups. Participants were divided into four subgroups according to GAD-7 scores (≤4 vs. >4) and MIP-1α levels relative to the median within each anxiety symptom group. Violin areas represent data density, jittered points represent individual observations, and boxplots indicate the median and interquartile range. Vertical jittering was applied only to reduce point overlap; the original NIHSS scores were integer values. GAD, Generalized Anxiety Disorder Scale; NIHSS, National Institutes of Health Stroke Scale; MIP-1α, Macrophage Inflammatory Protein-1α.

Serum MCP-1 and MIP-1β concentrations also differed significantly across the four groups (H = 34.11 and 130.66, respectively; both p < 0.001). *Post-hoc* analyses showed that the two MIP-1α ≥median groups had significantly higher MCP-1 and MIP-1β concentrations than the two MIP-1α <median groups (all adjusted p < 0.001). No significant differences were observed between the two anxiety symptom groups within the same MIP-1α stratum.

### 3.2 Correlation analyses of clinical and biochemical indicators

**Figure 2** presents the Spearman rank correlation matrix of clinical and biochemical variables stratified by GAD-7 (≤ 4 [lower anxiety symptom group] vs. > 4 [higher anxiety symptom group]). Correlations for the GAD-7 ≤ 4 subgroup are shown in the lower triangle, whereas those for the GAD-7 > 4 subgroup are shown in the upper triangle.

**Figure 2:**
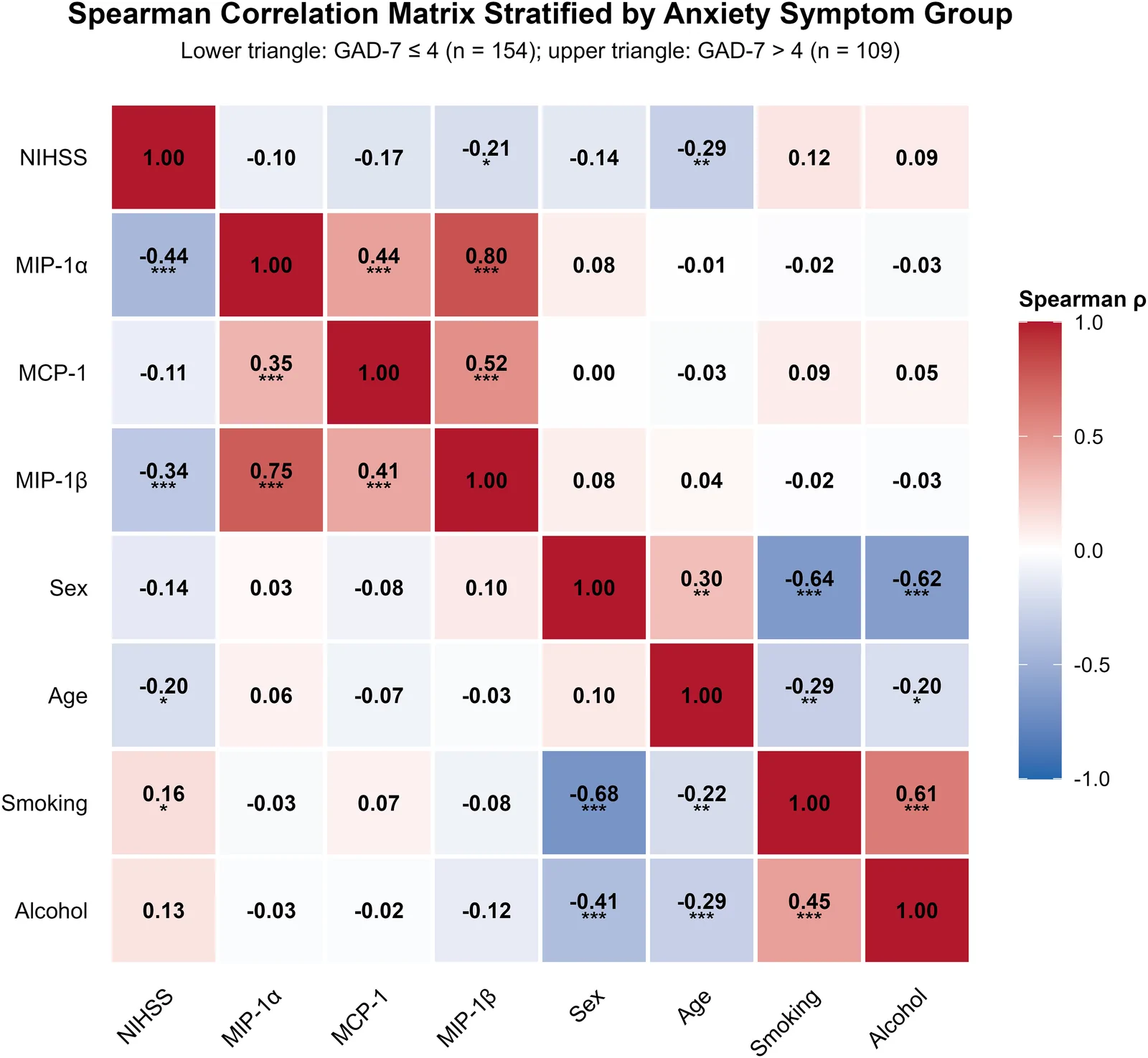
Correlation matrix of clinical and biochemical indicators stratified by GAD-7 (≤4 vs. >4). The lower triangle (below the diagonal) corresponds to the group with GAD-7 ≤4, and the upper triangle (above the diagonal) corresponds to the group with GAD-7 >4. Values are Spearman’s rank correlation coefficients. *p < 0.05, **p < 0.01, ***p < 0.001. GAD, Generalized Anxiety Disorder Scale; NIHSS, National Institutes of Health Stroke Scale; MIP-1α, Macrophage Inflammatory Protein-1α; MCP-1, Monocyte Chemoattractant Protein-1; MIP-1β, Macrophage Inflammatory Protein-1β.

MCP-1 and MIP-1β demonstrated consistent correlation patterns across both anxiety symptom severity strata, showing positive correlations with NIHSS scores in both the lower anxiety symptom group and higher anxiety symptom group. In contrast, MIP-1α exhibited a distinct, anxiety symptom severity dependent reversal in its association with stroke severity. Specifically, MIP-1α was significantly negatively correlated with NIHSS scores in the GAD-7 ≤ 4 subgroup, whereas this relationship shifted to a positive correlation in the GAD-7 > 4 subgroup. Additionally, MIP-1α was strongly and positively correlated with MCP-1 and MIP-1β in both subgroups (all p < 0.001), consistent with coordinated expression of inflammatory chemokines.

Demographic and lifestyle variables, including age, sex, smoking status, and alcohol consumption, showed weak or non-significant correlations with inflammatory markers and NIHSS scores and did not materially influence the primary correlation patterns observed.

### 3.3 Moderating effect of anxiety symptom severity on the association between MIP-1α and NIHSS scores

Based on the anxiety symptom group-stratified correlation patterns observed in **Figure 2**, an exploratory, hypothesis-generating analysis was performed to examine whether anxiety symptom group moderated the association between MIP-1α and stroke severity. Results from the hierarchical linear regression models are presented in **Table 2**.

**Table 2:** Linear regression analysis for the moderation effect of GAD on the relationship between MIP1α and NIHSS in total stroke patients.

	Mode 1 (NIHSS)		Model 2 (NIHSS)		Model 3 (NIHSS)	
	Standardized β	t value	Standardized β	t value	Standardized β	t value
Age	−0.18**	−2.81	−0.20**	−3.15	−0.19**	−3.09
Sex	−0.02	−0.19	−0.05	−0.62	−0.06	−0.69
Smoking	−0.01	−0.07	0.02	0.19	0.01	0.17
Drinking	0.02	0.31	−0.01	−0.12	−0.01	−0.14
Diabetes	0.07	1.21	0.07	1.25	0.07	1.19
Hypertension	−0.07	−1.20	−0.07	−1.15	−0.06	−1.07
GAD-7 group	—	—	0.24***	3.92	0.24***	3.97
MIP-1α	—	—	−0.18**	−3.10	−0.19**	−3.28
MIP-1α × GAD-7	—	—	—	—	0.16**	2.75
Adjusted R²	0.02		0.10***		0.13***	
F	1.96		4.80		5.22	

All data were centered and standardized prior to analysis. Anxiety symptom group was coded as 0 for GAD-7 ≤4 and 1 for GAD-7 >4. Model 1 was a linear regression model including Age, Sex, Smoke and Drink as the independent variables, with NIHSS as the dependent variable. Model 2 was another linear regression model that included GAD and MIP1α as the inde- pendent variables, with NIHSS as the dependent variable. Model 3 included GAD, MIP1α, GAD×MIP1α terms as independent variables and NIHSS as the dependent variable. Both Model 2 and the Model 3 were adjusted for Age, Sex, Smoke and Drink. **p <0.01, ***p <0.001.

GAD, Generalized Anxiety Disorder Scale; NIHSS, National Institutes of Health Stroke Scale; MIP-1α, Macrophage Inflammatory Protein-1α; MCP-1, Monocyte Chemoattractant Protein-1; MIP-1β, Macrophage Inflammatory Protein-1β.

After adjustment for age, sex, smoking, alcohol consumption, hypertension, and diabetes, MIP-1α remained negatively associated with NIHSS scores (β = −0.19, p = 0.001). The interaction between MIP-1α and anxiety symptom group was significant (β = 0.16, p = 0.006), indicating that the inverse association between MIP-1α and NIHSS scores was attenuated in the higher anxiety symptom group. Age was inversely associated with NIHSS scores (β = −0.19, p = 0.002), whereas sex, smoking, alcohol consumption, hypertension, and diabetes were not significantly associated with NIHSS scores (all p > 0.05).

The conceptual moderation model is presented in **Figure 3A**, and the corresponding simple slope analysis is shown in **Figure 3B**. MIP-1α was significantly and inversely associated with NIHSS scores in the lower anxiety symptom group (GAD-7 ≤4; β = −0.32, p < 0.001), whereas no association was observed in the higher anxiety symptom group (GAD-7 >4; β = −0.003, p = 0.969). These findings support an anxiety symptom group-dependent difference in the association between MIP-1α and NIHSS scores.

**Figure 3:**
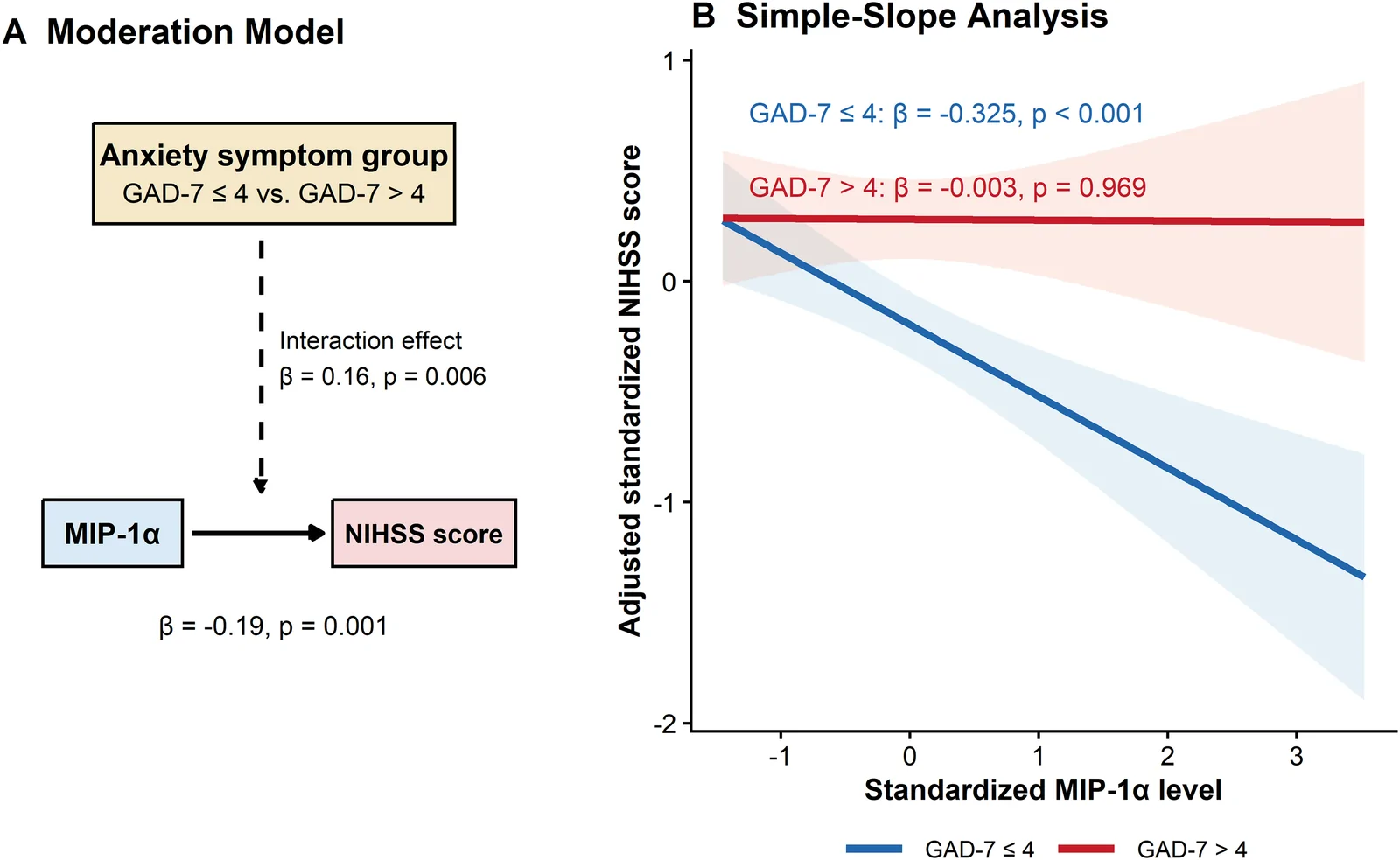
Moderation effect of anxiety symptom severity on the relationship between MIP-1α and NIHSS total scores in total stroke patients. **(A)** Conceptual model of hypothesis-generating analysis regarding anxiety symptom severity as moderator. **(B)** Simple slope analysis for the moderation effect of anxiety symptom severity on the relationship between MIP1α and NIHSS total scores. The model was adjusted by Age, Sex, Smoke and Drink. The lower and higher anxiety symptom groups were defined as GAD-7 ≤4 and GAD-7 >4, respectively. GAD, Generalized Anxiety Disorder Scale; NIHSS, National Institutes of Health Stroke Scale; MIP-1α, Macrophage Inflammatory Protein-1α.

### 3.4 Predictive performance of regression models (AUC analysis)

**Figure 4** illustrates the receiver operating characteristic (ROC) curves for predicting NIHSS scores based on the three regression models. Sensitivity (true positive rate) is plotted against 1 − specificity (false positive rate). The areas under the ROC curves (AUCs) for Models 1, 2, and 3 were 0.67, 0.74, and 0.76, respectively, showing a numerical increase in discriminatory performance across the three models with the sequential inclusion of anxiety symptom severity, MIP-1α levels, and their interaction term.

**Figure 4:**
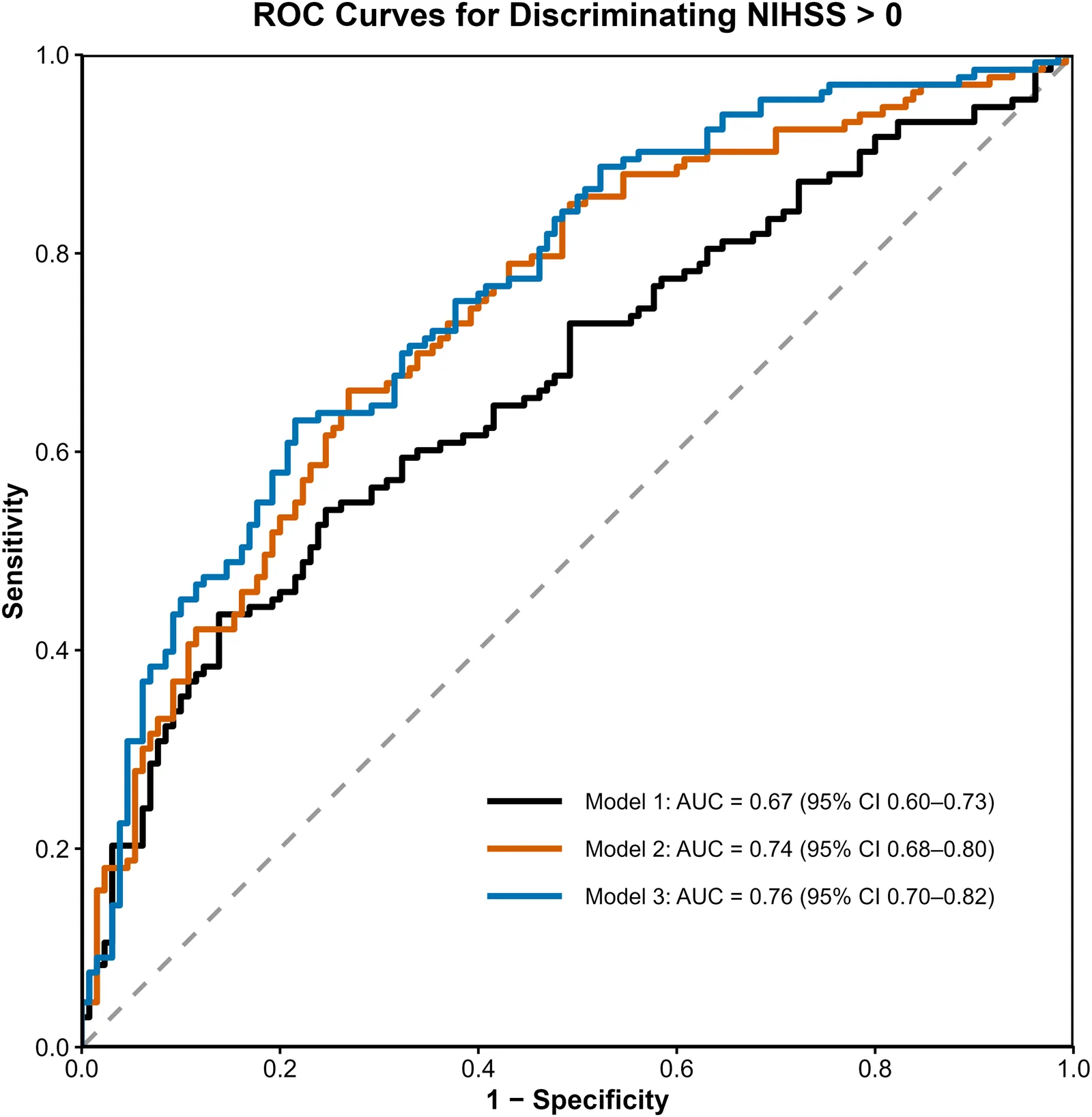
Receiver operating characteristic (ROC) curves for predicting NIHSS scores across three Models. Sensitivity (y-axis) represents the true positive rate, and 1 − specificity (x-axis) represents the false positive rate. ROC analyses used a binary outcome of NIHSS = 0 versus NIHSS >0. NIHSS, National Institutes of Health Stroke Scale.

**Table 3** summarizes the DeLong pairwise comparisons of the AUCs among the three models. Model 2 showed significantly greater discrimination than Model 1 (Z = −2.70, p < 0.05), and Model 3 significantly outperformed Model 1 (Z = −3.41, p < 0.001). However, Model 3 did not significantly outperform Model 2 (Z = −1.48, p = 0.14), indicating that the interaction term did not provide a statistically significant incremental gain in discrimination beyond the main effects.

**Table 3:** DeLong’s test for comparing AUCs of three ROC curves.

Model comparison	AUC (model 1)	AUC (model 2)	AUC (model 3)	95% CI		Z-score	P-value
				Lower	Upper		
Model 1 vs Model 2	0.67	0.74	–	-0.12	-0.02	-2.70	<0.01**
Model 1 vs Model 3	0.67	–	0.76	-0.15	-0.04	-3.41	<0.001***
Model 2 vs Model 3	–	0.74	0.76	-0.05	0.01	-1.48	0.14

DeLong’s test was used to compare the area under the ROC curve (AUC) of three models predicting NIHSS scores. The binary outcome for ROC analysis was NIHSS >0 versus NIHSS = 0. **p < 0.01, ***p < 0.001.

95% CI, 95% confidence interval.

## 4 Discussion

To our knowledge, this study provides the first clinical evidence demonstrating a significant moderating effect of anxiety symptom severity on the association between the chemokine macrophage inflammatory protein-1α (MIP-1α) and ischemic stroke (IS) severity. This central finding highlights an active role of post-stroke anxiety symptoms (PSAS) in acute-phase stroke pathophysiology, extending beyond the traditional view of anxiety as a mere consequence of neurological injury and instead revealing a complex, reciprocal interaction between neuroinflammatory processes and psychological stress.

The cross-sectional design of this study precludes any inferences regarding causality, temporal sequence, or underlying biological mechanisms. The observed moderation effect reflects statistical interaction rather than proven biological causation. Longitudinal studies with serial biomarker and symptom tracking are necessary to clarify directionality.

Hierarchical regression analyses clearly indicate that the impact of MIP-1α on stroke severity is condition dependent, with both the direction and magnitude of its effect contingent upon anxiety status. Specifically, in patients with elevated anxiety levels (GAD-7 > 4), higher MIP-1α concentrations were associated with a trend toward increased NIHSS scores, whereas in patients with low anxiety levels (GAD -7 ≤ 4), MIP-1α exhibited a weak inverse or potentially protective association with neurological impairment. This divergence suggests a context-dependent, dual role of MIP-1α across distinct neurobiological states(PMID: 29405771, PMID: 37026459). In the absence of heightened psychological stress, MIP-1α may preferentially participate in adaptive processes such as neuroplasticity, immune regulation, or tissue repair. In contrast, we hypothesize that an acute anxiety state could theoretically interact with hypothalamic–pituitary–adrenal (HPA) axis signaling, which might alter neuroinflammatory responses. It should be emphasized that the potential biological pathways discussed herein—including stress-induced HPA-axis hyperactivity, upregulation of chemokine receptors (CCR1/CCR5), and downstream blood-brain barrier disruption—are working hypotheses inferred from previous literature rather than direct empirical evidence measured in the current study. Because our biomarker and psychiatric evaluations were conducted cross-sectionally, these mechanisms require direct validation in mechanistic animal models and prospective clinical trials to avoid over-interpreting correlation as proven biological causality. However, because our study assessed these markers concurrently, this biological mechanism remains a working hypothesis that warrants prospective investigation (16), thereby disrupting blood–brain barrier integrity and potentiating MIP-1α–mediated neuroinflammatory signaling (17), ultimately exacerbating neuronal injury and neurological deficits. This apparent contradiction aligns with the dual biological role of CC-chemokines and their receptors in stroke pathophysiology. Under low baseline stress or physiological conditions, MIP-1α signaling contributes to homeostatic phagocytosis, tissue clearance, and neurovascular repair. However, in the presence of heightened psychological distress such as anxiety, stress-induced HPA-axis hyperactivity may prime the neuroimmune microenvironment, converting MIP-1α signaling into a detrimental, hyper-inflammatory response that exacerbates blood-brain barrier failure and neurological deficits (18).

An inverse association between age and NIHSS scores was also observed. Given the restricted inclusion of patients with mild-to-moderate stroke, this finding may reflect selection and range-restriction effects rather than a protective association of older age and should therefore be interpreted cautiously.

Recent clinical studies in stroke cohorts further confirm that circulating CC-chemokine dysregulation is closely correlated with post-stroke anxiety symptoms and functional recovery in human patients (8, 19). Notably, accumulating evidence indicates that stress-related mediators upregulate the expression of CC chemokine receptors 1 and 5 (CCR1 and CCR5), the principal receptors for MIP-1α, on both peripheral monocytes and central microglia (19–21). Such receptor upregulation may “prime” the neuroimmune microenvironment by lowering the activation threshold for MIP-1α–dependent signaling cascades, including the mitogen-activated protein kinase (MAPK) and nuclear factor-κB (NF-κB) pathways (22). Through this psychological-to-biological amplification loop, anxiety may intensify neuroinflammatory responses, further compromise blood–brain barrier integrity, and ultimately worsen neurological outcomes. These findings support the notion that anxiety is not merely an epiphenomenon of cerebral injury but a biologically anxiety symptoms what are associated with altered relationships between MIP-1α and stroke severity that substantially shapes the effects of inflammatory mediators on the injured brain.

ROC analysis showed that Model 3 had significantly greater discriminatory performance than Model 1 (p <0.001). However, Model 3 did not significantly outperform Model 2 (p = 0.14), indicating that the interaction term did not provide a statistically significant incremental gain in discrimination beyond the main effects of anxiety symptoms and MIP-1α. The statistically significant interaction and the absence of a significant incremental improvement in AUC are not contradictory. The interaction indicates that the association between MIP-1α and NIHSS scores differs according to anxiety symptom group, whereas the AUC reflects the model’s overall ability to discriminate between patients with NIHSS = 0 and those with NIHSS > 0. Thus, although the interaction effect was statistically detectable, its effect size was modest and insufficient to produce a significant improvement in discrimination in the present sample. Larger independent cohorts are required to validate its reproducibility and practical clinical utility.

Building on the observed moderation findings, this study offers important implications for clinical translation. First, the prognostic utility of MIP-1α as a biomarker should be interpreted in the context of patients’ concurrent anxiety status to enable truly precise risk stratification. Second, the identified moderation effect highlights a promising precision target for therapeutic intervention. Given the adverse association between elevated MIP-1α levels and NIHSS scores among patients with high anxiety, targeted strategies for this high-risk subgroup, such as early identification and management of anxiety symptoms, or the combined use of interventions aimed at attenuating MIP-1α–mediated neuroinflammation, may represent effective combinatorial approaches for improving neurological outcomes. Accordingly, future clinical trials should prioritize evaluating the efficacy and safety of such integrated interventions in stroke populations characterized by heightened anxiety.

Several limitations should be considered. First, the cross-sectional design precludes conclusions regarding causality, temporal sequence, or underlying biological mechanisms. Second, this single-center study included only patients with mild-to-moderate ischemic stroke, with a median NIHSS score of 1, which limits generalizability to patients with severe or hemorrhagic stroke. Third, anxiety symptoms were assessed using the self-reported GAD-7 without a structured diagnostic interview. Therefore, the findings reflect anxiety symptom severity rather than clinically diagnosed anxiety disorders and may be affected by reporting bias during the acute phase. Fourth, MIP-1α was measured at a single time point and may not fully reflect the temporal dynamics of the inflammatory response. Finally, although the models were adjusted for age, sex, smoking, alcohol consumption, hypertension, and diabetes, exact onset-to-assessment intervals, reperfusion treatment, infarct location and volume, stroke subtype, previous stroke, medication use, and social support were not recorded. Residual confounding by these factors therefore cannot be excluded. Furthermore, the potential for reverse causality cannot be ruled out due to our cross-sectional design. It is plausible that baseline stroke severity directly drives both acute systemic neuroinflammation and psychological distress simultaneously, wherein severe brain ischemia elevates MIP-1α levels while independently triggering post-stroke anxiety symptoms. Future prospective longitudinal studies with serial measurements are essential to disentangle these dynamic bidirectional causality paths.

To address these limitations, future studies should prioritize longitudinal designs to characterize the temporal co-evolution of post-stroke anxiety symptoms and inflammatory biomarkers. Further mechanistic investigations are also needed to elucidate how hypothalamic–pituitary–adrenal axis dysregulation and blood–brain barrier permeability interact to amplify chemokine-mediated neurotoxicity. Ultimately, well-designed prospective clinical trials are required to determine whether integrated treatment strategies, combining early anxiety management with targeted anti-inflammatory therapies, can synergistically improve outcomes in stroke survivors with elevated anxiety levels.

## Data Availability

The raw data supporting the conclusions of this article will be made available by the authors, without undue reservation.
